# Separation Surgery, Fixation With Carbon-Fiber Implants, and Stereotactic Body Radiotherapy for Oligometastatic Spinal Disease

**DOI:** 10.7759/cureus.31370

**Published:** 2022-11-11

**Authors:** Richard Galloway, Nicholas Gikas, Ramez Golomohammad, Jenny Sherriff, Marcin Czyz

**Affiliations:** 1 Sarcoma and Joint Reconstruction Unit, Royal National Orthopaedic Hospital, London, GBR; 2 Neurological Surgery, University Hospital Coventry and Warwickshire, Coventry, GBR; 3 Orthopaedics, St. George’s Hospital, London, GBR; 4 Oncology, Queen Elizabeth Hospital Birmingham, Birmingham, GBR; 5 Neurological Surgery, Queen Elizabeth Hospital Birmingham, Birmingham, GBR

**Keywords:** carbon fibre implants, separation surgery, stereotactic radiosurgery, breast cancer, spinal metastasis

## Abstract

The management of spinal metastases focuses on reducing symptoms and protecting the spinal cord, historically involving extracorporeal radiotherapy alone. The use of separation surgery techniques alongside high-dose radiotherapy to treat spinal metastases is a novel concept and has changed the treatment paradigm. Additionally, titanium implants have been increasingly used in cases of metastatic spinal tumours requiring adjuvant stereotactic radiotherapy (SBRT). We present the case of a 48-year-old female patient who was diagnosed with a metastatic deposit of breast cancer within L1 with an Epidural Spinal Cord Compression score greater than 1a. At the time of the diagnosis, her prognosis was estimated to be more than two years. She underwent a posterior instrumented fusion of T11-L3 vertebrae with a carbon-fibre fixation system and separation surgery (debulking of the tumour around the spinal cord). The patient was discharged on the second postoperative day achieving complete resolution of the mechanical back pain. SBRT was performed 12 weeks after the surgery. The patient regained ECOG status of 1 shortly after but sadly passed away due to multiple brain metastases 36 months following posterior fixation. Her spinal disease remained well-controlled throughout the follow-up.

Carbon-fibre implants appear to be safe and relatively easy to apply. Their use, due to limited artefacts in both computed tomography and magnetic resonance imaging, makes SBRT much more straightforward and follow-up imaging easier to be interpreted. Our experience demonstrates that, in conjunction with separation surgery, the translucent, low perturbing properties of these implants can improve SBRT intervention and detection of recurrence on follow-up imaging.

## Introduction

The management of spinal metastases focuses on reducing symptoms and protecting the spinal cord, historically involving extracorporeal radiotherapy alone [1]. The use of separation surgery techniques alongside high-dose radiotherapy to treat spinal metastases has increased. There has been an ongoing debate regarding whether non-titanium implants should be used in cases of metastatic spinal tumours requiring adjuvant stereotactic radiotherapy (SBRT). We present the case of a 48-year-old female patient who was diagnosed with a metastatic deposit of breast cancer within L1. She underwent a posterior instrumented fusion with a carbon-fibre fixation system and separation surgery (debulking of the tumour around the spinal cord), with subsequent SBRT therapy (CyberKnife®, Sunnyvale, CA, USA).

This article was previously presented as a meeting poster at the 2022 AO Global Spinal Congress on June 1, 2022.

## Case presentation

Background

A 48-year-old female patient, with a Karnofsky Performance Score of 80, was referred to our tertiary centre due to lytic oligo-metastasis within the vertebral body L1 causing ongoing mechanical pain and discomfort, with no signs of compression to the neural structures. A single, small, and asymptomatic deposit within the right iliac crest was identified. The patient previously underwent a right mastectomy and axillary node clearance for grade 3 breast carcinoma two years earlier. During the mastectomy, a non-magnetic resonance (MR) compatible breast expander was used. Computed tomography (CT) of the entire spine, bone scan, and positron emission tomography-computed tomography (PET-CT) were available at the time of presentation. Pre-procedure it was decided, after discussion with the patient and the multidisciplinary team, that the MR incompatible breast implants should be removed to aid with imaging. Based on radiological analysis of the preoperative imaging, the Epidural Spinal Cord Compression (ESCC) score was estimated to be greater than 1a as there were signs of deformation of the theca, demonstrating its close proximity to the spinal cord.

Diagnostic imaging

**Figure 1:**
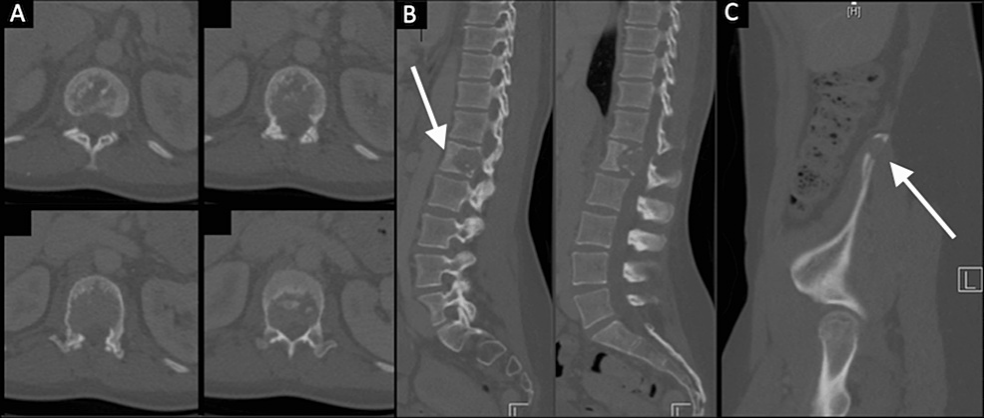
Preoperative computed tomography scan of the lumbosacral spine and pelvis. A: axial views showing a significant amount of lysis to the vertebral body and both pedicles of L1 with some volume of the epidural disease. B: sagittal cuts demonstrating the extent of the involvement of the anterior column and early signs of pathological fracture to both superior and inferior endplates. C: small lytic lesion reported as likely to represent a metastasis with the right iliac crest. This was felt to be accessible for stereotactic radiotherapy and treated subsequently.

A CT of the thorax abdomen pelvis (TAP), 18 days prior to the operation, showed soft-tissue attenuation with irregular margins at the L1 vertebral body and the right iliac crest, consistent with metastatic deposits. Further analysis identified areas of cortical bone destruction, its appearance harmonious with a coronally oriented pathological fracture of L1 involving both the middle and much of the anterior column (Figure 1).

**Figure 2:**
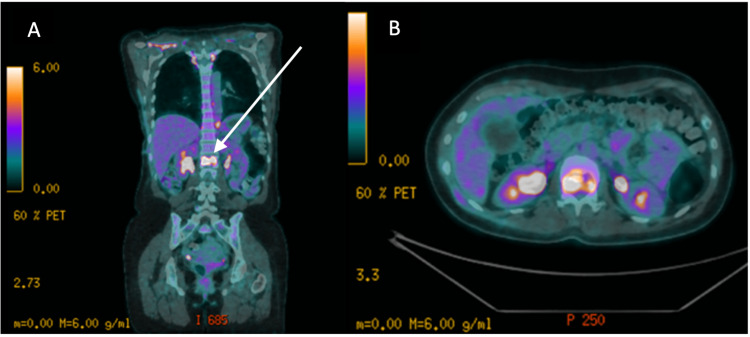
Positron emission tomography-computed tomography findings. Scan showing metabolically avid lesion within the vertebral body L1 concordant with the report of the plain computed tomography. There were no other soft-tissue or bony metastases revealed apart from the aforementioned small iliac crest deposit. A: coronal section; B: axial cut at the level of L1.

MRI revealed encroachment of the theca with no major compression of neural structures. Fluorodeoxyglucose positron emission tomography (FDG-PET) was performed and showed high-grade metabolically active lytic regions at the L1 vertebral body as well as the right iliac crest. These regions displayed additional focus, characteristic of metastatic deposits (Figure 2).

Bone scintigraphy delineated peripheral increased activity within the L1 vertebral body which correlated to the osteoblastic activity around the large lytic lesion seen on the CT scan. The right iliac crest lytic lesion was not visualised on the bone scan.

Procedure

**Figure 3:**
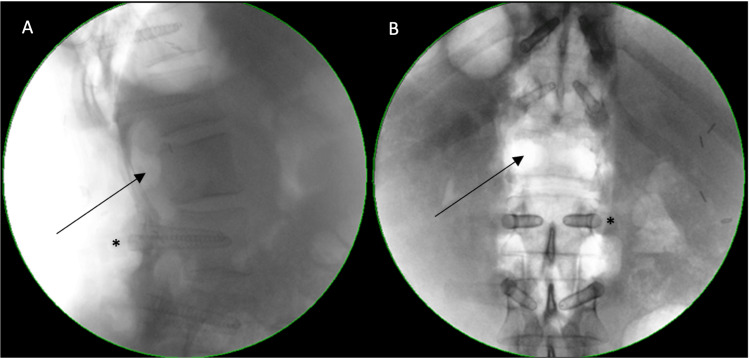
Intraoperative fluoroscopy. Radiolucent pedicle screw made of carbon fibre and coated with a 0.1 mm layer of titanium to ensure intraoperative visualisation marked with the asterisks. Complete bilateral pediculectomy can be visualised with the arrows. A: lateral view; B: anteroposterior view.

The patient was positioned prone on padded bolsters under general anaesthesia. The vertebral metastases and pathological fracture of the L1 vertebral body were managed with a posterior instrumented fusion (T11 to L3) and decompression of the L1 level. A standard midline approach was performed exposing the posterior aspect of the T11 to L3 vertebrae. Fusion using bone allograft with demineralised bone matrix (DBX, DuPuy) was performed. Free-hand instrumentation of the T11, T12, L2, and L3 levels was performed and the position of the screws was verified with an intraoperative X-ray. A wide laminectomy at T12-L2 was performed. The distended epidural veins were coagulated, and a large bulk of the tumour was removed from the vertebral body and epidural space. The posterior longitudinal ligament was transected at the level of discs T12/L1 and L1/2 and specimens were taken for pathology. Satisfactory decompression of the neural structures and no intraoperative complications were noted. Final tightening of the screws over the pre-contoured carbon-fibre rod was performed. High-volume local anaesthetic (Naropin - 7.5 mg mixed with sodium chloride) was injected into the muscles and skin before closing. The wound was closed in the standard fashion with 1 g of vancomycin powder spread in the deep layers of the wound. The patient subsequently received SBRT (CyberKnife®, Sunnyvale, CA, USA) 27 Gy in three alternate-day fractions to L1 and 30 Gy in three alternate-day fractions to the right iliac crest 12 weeks following the surgical procedure. Intraoperative fluoroscopy can be appreciated in Figure 3.

Outcome and follow-up

**Figure 4:**
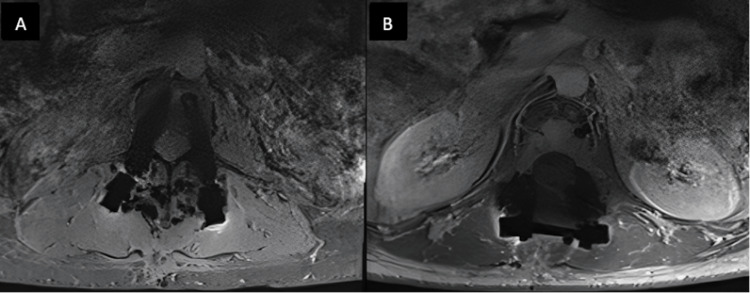
Postoperative magnetic resonance imaging scan obtained prior to stereotactic radiotherapy. T1-weighted images with intravenous gadolinium contrast. A: axial cut at the level of T12. Note carbon-fibre screws with minimal artefacts and good visibility of the content of the spinal canal. B: axial cut at the level of L1 with enhancing postoperative changes within the resection cavity and no compression of the theca, facilitating safe delivery of stereotactic radiotherapy.

**Figure 5:**
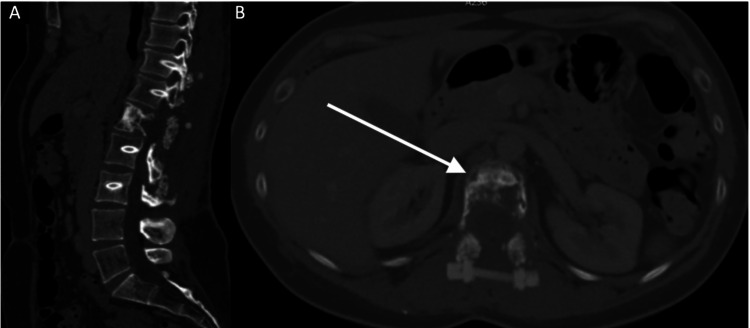
Postoperative computed tomography scan obtained 10 months following the stereotactic radiotherapy. A: sagittal reconstruction showing the calcified ventral aspect of the vertebral body L1 and no active metastatic disease. B: axial cut at the level of L1 showing calcification with the irradiated tumour and carbon-fibre implants. Note the complete lack of artefacts.

This patient underwent an uncomplicated procedure and was discharged on the second postoperative day, quickly regaining mobility. Follow-up imaging revealed adequate decompression of the spinal cord, facilitating high-dose radiotherapy (Figures 4, 5).

The patient adhered to their radiosurgery plan with the oncologist, who reported an excellent response to the SBRT with no reported complications. There was no subsequent spinal cord compression, and her symptomatic back pain was reduced from a visual analogue scale (VAS) score of 10 preoperatively to 2 at the six-month follow-up. Further postoperative imaging showed a decrease in the size of both the oligometastases at L1 and the right iliac crest, with stable control and no signs of progression over 36 months of follow-up. She subsequently died from effects relating to multiple metastatic brain deposits; however, she remained pain-free and comfortable regarding her spinal disease throughout.

## Discussion

Traditional management

The management of spinal metastases focuses on reducing symptoms and protecting the spinal cord, historically involving extracorporeal radiotherapy alone. SBRT has been a major innovation in the treatment of spinal metastases [2,3]. SBRT allows for focused delivery of high-dose radiotherapy, accurately treating spinal tumours, and reducing collateral damage to adjacent structures. Garcia-Barros et al. found that it works by increasing a signal transduction platform within the cell, resulting in microvascular endothelial dysfunction and apoptosis [4]. Multiple studies have demonstrated that SBRT produces clinical benefits in spinal metastases, with both symptomatic relief and superior local control of cancer [5-7]. This technique is increasingly successful, partly due to improvements in non-invasive patient immobilisation, alongside more advanced image-guided radiation delivery systems. Current evidence points to the excellent results from SBRT; however, as with all radiotherapy, there remains the risk of toxicity. There must be a careful balance between underdosing patients and damaging adjacent structures [8]. The most critical structure is the spinal cord which is especially sensitive to high-dose radiation. Gibbs et al. found that there was a 0.5% risk of myelopathy at an 8 Gy equivalent dose [9]. Sahgal et al. found that myelopathy was reported in single fractions of 10.6 Gy and suggested that current guidelines of 10 Gy per fraction should be respected [10].

Traditional techniques for spinal stabilisation use titanium implants. The high atomic number of the metal within these implants reduces the radiation dose forwarded to the underlying tumour and can present as an artefact on imaging. The unwanted image artefacts are created due to beam hardening, scatter, and noise, complicating the interpretation of follow-up imaging and causing the precise dose calculation to be inaccurate [11,12]. In some series, the presence of metallic hardware was the strongest predictive factor for local recurrence in spinal tumours treated with particle therapy [13].

Our management and rationale

Our patient underwent a tumour separation procedure with instrumented fusion, carbon-fibre implants and postoperative SBRT. Due to two metastatic lesions revealed by imaging, we deemed an en-bloc resection of the spinal lesion inappropriate. After the removal of MR incompatible breast expanders, imaging demonstrated the ESCC to be greater than 1a, and it was decided that adequate SBRT would be related to an unacceptably high risk of toxicity to the spinal cord if performed without separation surgery.

The concept of hybrid therapy with separation surgery and SBRT as a treatment for spinal metastases has been discussed previously [1]. Separation surgery refers to the stabilisation and circumferential decompression of the thecal sac and nerve roots by resecting the posterior longitudinal ligament. The goal is to provide an ablative subject for SBRT within the spinal cord constraints. Laufer et al. reflect on the importance of providing appropriate levels of radiation to the tumour for successful treatment while acknowledging the toxic effects of radiation to the spinal cord [14]. By creating a space between the tumour and the spinal cord, we can safely and effectively provide adequate dosages of radiotherapy. Multiple studies have shown that by providing high-dose SBRT in fewer fractions, the local progression rate is decreased [15,16]. Tumour separation with instrumented fusion and postoperative SBRT was discussed and considered appropriate by the multidisciplinary spinal oncology team which involved surgeons, clinical and radiation oncologists, as well as histopathologists. A tumour separation procedure (resection of the tumour volume from the epidural space potentially in contact with the spinal cord) with the posterior instrumented fusion of levels T11- L3 was performed.

To facilitate SBRT treatment and follow-up, the Carbon-Fibre Reinforced Polyetheretherketone (CFR-PEEK, also referred to as CarboFix implant system [Carboclear, Israel]) was used. The use of CFR-PEEK materials to remedy the problems caused by titanium implants has been trialled before in spinal surgery. This is due to the material’s biocompatibility, its ability to promote bone fusion, and its low modulus of elasticity which closely matches that of bone [17]. It has been suggested that CFR-PEEK fixation systems (including rods and screws) may have a useful role in spinal metastatic disease procedures with the potential to make postoperative radiotherapy easier and more effective [18]. It has been shown that carbon-fibre implants perturb radiation doses far less than metallic implants [19]. This increases the proportion of radiation forwarded through to the tumour site and decreases the irradiation of neighbouring structures due to decreased scattering of particles. Furthermore, the reduction of artefacts on imaging aids with accurate localisation of the radiotherapy beam, reducing inappropriate irradiation of surrounding tissues. Moreover, the radiolucent property of the CFR-PEEK implants aids the detection of local recurrence on follow-up imaging. Boriani et al. found that the lack of artefacts on imaging resulted in early local recurrence detection and improved oncologist planning [20]. In addition, the CFR-PEEK implants were comparable to the standard titanium system in terms of intraoperative complications, stability at weight bearing, and at functional recovery.

## Conclusions

Separation surgery may facilitate efficient and safe SBRT in appropriately selected cases of spinal oligometastatic disease. Carbon-fibre implants appear to be safe and relatively easy to utilise intraoperatively. Our experience demonstrates that, when used alongside separation surgery, the translucent, low perturbing properties of these implants can improve SBRT intervention and detection of recurrence on follow-up imaging. Any clinical guidelines and recommendations regarding this treatment modality need to be based on a thorough analysis of constantly emerging data.

## References

[1] Di Perna G, Cofano F, Mantovani C (2020). Separation surgery for metastatic epidural spinal cord compression: a qualitative review. J Bone Oncol.

[2] Alongi F, Arcangeli S, Filippi AR, Ricardi U, Scorsetti M (2012). Review and uses of stereotactic body radiation therapy for oligometastases. Oncologist.

[3] Chang BK, Timmerman RD (2007). Stereotactic body radiation therapy: a comprehensive review. Am J Clin Oncol.

[4] Garcia-Barros M, Paris F, Cordon-Cardo C (2003). Tumor response to radiotherapy regulated by endothelial cell apoptosis. Science.

[5] Yamada Y, Katsoulakis E, Laufer I (2017). The impact of histology and delivered dose on local control of spinal metastases treated with stereotactic radiosurgery. Neurosurg Focus.

[6] Yamada Y, Lovelock DM, Yenice KM, Bilsky MH, Hunt MA, Zatcky J, Leibel SA (2005). Multifractionated image-guided and stereotactic intensity-modulated radiotherapy of paraspinal tumors: a preliminary report. Int J Radiat Oncol Biol Phys.

[7] Gerszten PC, Burton SA, Ozhasoglu C, Welch WC (2007). Radiosurgery for spinal metastases: clinical experience in 500 cases from a single institution. Spine (Phila Pa 1976).

[8] Sahgal A, Weinberg V, Ma L (2013). Probabilities of radiation myelopathy specific to stereotactic body radiation therapy to guide safe practice. Int J Radiat Oncol Biol Phys.

[9] Gibbs IC, Patil C, Gerszten PC, Adler JR Jr, Burton SA (2009). Delayed radiation-induced myelopathy after spinal radiosurgery. Neurosurgery.

[10] Sahgal A, Ma L, Gibbs I (2010). Spinal cord tolerance for stereotactic body radiotherapy. Int J Radiat Oncol Biol Phys.

[11] Krätzig T, Mende KC, Mohme M (2021). Carbon fiber-reinforced PEEK versus titanium implants: an in vitro comparison of susceptibility artifacts in CT and MR imaging. Neurosurg Rev.

[12] Zimel MN, Hwang S, Riedel ER, Healey JH (2015). Carbon fiber intramedullary nails reduce artifact in postoperative advanced imaging. Skeletal Radiol.

[13] Rutz HP, Weber DC, Sugahara S (2007). Extracranial chordoma: outcome in patients treated with function-preserving surgery followed by spot-scanning proton beam irradiation. Int J Radiat Oncol Biol Phys.

[14] Laufer I, Bilsky MH (2019). Advances in the treatment of metastatic spine tumors: the future is not what it used to be. J Neurosurg Spine.

[15] Moulding HD, Elder JB, Lis E, Lovelock DM, Zhang Z, Yamada Y, Bilsky MH (2010). Local disease control after decompressive surgery and adjuvant high-dose single-fraction radiosurgery for spine metastases. J Neurosurg Spine.

[16] Laufer I, Iorgulescu JB, Chapman T (2013). Local disease control for spinal metastases following "separation surgery" and adjuvant hypofractionated or high-dose single-fraction stereotactic radiosurgery: outcome analysis in 186 patients. J Neurosurg Spine.

[17] Kersten RF, van Gaalen SM, de Gast A, Öner FC (2015). Polyetheretherketone (PEEK) cages in cervical applications: a systematic review. Spine J.

[18] Hak DJ, Mauffrey C, Seligson D, Lindeque B (2014). Use of carbon-fiber-reinforced composite implants in orthopedic surgery. Orthopedics.

[19] Nevelsky A, Borzov E, Daniel S, Bar-Deroma R (2017). Perturbation effects of the carbon fiber-PEEK screws on radiotherapy dose distribution. J Appl Clin Med Phys.

[20] Boriani S, Tedesco G, Ming L (2018). Carbon-fiber-reinforced PEEK fixation system in the treatment of spine tumors: a preliminary report. Eur Spine J.

